# Concomitant sexually transmitted diseases in patients with newly diagnosed HIV in Sri Lanka

**DOI:** 10.1186/1742-4690-9-S1-P84

**Published:** 2012-05-25

**Authors:** SD Dharmaratne, K Buddhakarale

**Affiliations:** 1 National STD AIDS Control Program, Sri Lanka

## Introduction

Diagnosing and treating STI are important both for the health of HIV infected persons, their sexual partners and for HIV prevention efforts. STI following an HIV diagnosis or STI co-infection can serve as a surrogate indicator of continued risk behaviors.

## Objective

To determine the prevalence of concomitant STI in newly diagnosed HIV positive patients.

## Method

Data was extracted from randomly selected individual clinical notes of patients who are attending the Central HIV clinic, Colombo.

## Results

A total of 187 subjects were included to the study of which 106 (57 %) were males and 80 (43 %) were females. The mean age of the sample was 37 years (SD =9.3) and the median CD4 count at diagnosis was 320 cells/μl. Routine STI screening had been done only in 90.3% (169) of the sample. Among the screened subjects for STIs, over one in every four (28%) had an STI. Ulcerative and non-ulcerative STIs were present in 18.2% and 10% of subjects respectively. Syphilis was positive in 9.5% (N=16), symptomatic Herpes simplex infection in 7.7% (N=13), non gonoccocal urithritis /cervicitis in 6.5% (N=11) and symptomatic genital warts in 3% (N=5). There was a statistically significant association between the number of life time partners and STI prevalence (P=0.011) and sexual orientation and STI prevalence (P=0.005). In contrast gender, marital status and age did not show any statistically significant association with STI prevalence.

## Conclusion

Significant numbers of HIV patients are co-infected with at least one ulcerative or non-ulcerative STI at the time of their HIV diagnosis which enhances HIV transmission. Therefore, careful and continued screening and management of STIs in HIV positive patients is an important HIV prevention intervention.

